# Apoptosis-Inducing Effect of Three Medicinal Plants on Oral Cancer Cells KB and ORL-48

**DOI:** 10.1155/2014/125353

**Published:** 2014-07-24

**Authors:** Mohd Zabidi Majid, Zuraiza Mohamad Zaini, Fathilah Abdul Razak

**Affiliations:** ^1^Department of Oral Biology and Biomedical Sciences, Faculty of Dentistry, University of Malaya, 50603 Kuala Lumpur, Malaysia; ^2^Department of Oro-Maxillofacial Surgical and Medical Sciences, Faculty of Dentistry, University of Malaya, 50603 Kuala Lumpur, Malaysia

## Abstract

*Brucea javanica, Azadirachta indica, *and *Typhonium flagelliforme* are medicinal plants commonly used to treat conditions associated with tumour formation. This study aimed to determine the antiproliferative activity of these plants extracts on KB and ORL-48 oral cancer cell lines and to suggest their mode of cell death. The concentration producing 50% cell inhibition (IC_50_) was determined and the activity was examined under an inverted microscope. Immunohistochemistry fluorescent staining method (TUNEL) was performed to indicate the mechanism of cell death and the fragmented DNA band pattern produced was obtained for verification. Compared to *Azadirachta* sp. and *Typhonium* sp., the antiproliferative activity of *Brucea* sp. extract was the most potent on both KB and ORL-48 cells with IC_50_ of 24.37 ± 1.75 and 6.67 ± 1.15 *µ*g/mL, respectively. Signs of cell attrition were observed 24 hr after treatment. Green fluorescent spots indicating cell death by apoptosis were observed in images of both cells following treatment with all the three extracts. DNA fragments harvested from *Brucea*-treated cells produced bands in a ladder pattern suggesting the apoptotic effect of the extract. It is thus concluded that *Brucea* sp. extract exhibited cytotoxic activity on ORL-48 cells and their action mechanism is via apoptosis.

## 1. Introduction

All mammalian cells carry a similar molecular machinery to regulate cell proliferation, differentiation, and death. Cancer cells have defects in these regulatory circuits that govern normal cell proliferation and homeostasis [1]. The ability of tumour cell populations to expand in number is determined by the rate of cell proliferation/cell death. A major source of cell death is apoptosis or programmed cell death. A cancerous cell acquires resistance against apoptosis.

Uncontrolled proliferation of cells in the oral mucosa may start as an abnormal cell overgrowth in the oral cavity. Often referred to as leukoplakia, a disorder normally presents itself as patches of white areas on the inner surface of the mouth which have been known to potentially become cancer [2]. There are no specific causes to explain the onset of such cancerous activities but one of the main characteristics of a cancer cell is the loss of controlled proliferative activity. Among the approaches to the treatment of cancer is the use of therapeutic agents and alternative medicine from plants in combination with surgery and/or radiotherapy. Intensive efforts to discover natural agents for the treatment of cancer had resulted from complaints on the many side effects experienced by cancer patients receiving radiotherapy and chemotherapy treatments. Traditional Chinese medicine (TCM) has shown success in treating various types of cancer. Among the many ingredients of TCM are the water extracts of* Lobelia chinensis*,* Rheum officinale* Baill,* Agrimoniapilosa* Ledeb.,* Sanguisorba officinalis* Linn., and* Paris polyphylla* Smith that are known to be effective in inhibiting the growth of human lung adenocarcinoma A549 and human breast cancer MCF-7 cells [3].

In the rain forest of Malaysia, many plants have been used in traditional preparations to prevent and treat various types of cancers.* Brucea* sp.,* Azadirachta* sp., and* Typhonium* sp. are three plants commonly used in folklore medicines in the Southeast Asia region. The active polar compounds of these plants are traditionally consumed in the form of a decoction [4].* Brucea* sp. is a plant under the family* Simaroubaceae*. The seeds of this plant have been reported to exhibit several biological activities including anticancer ones [5]. Due to its many bioactivities,* Brucea* sp. is used as a common ingredient in traditional Indonesian and Chinese medicines. According to Polonsky et al. [6], the active component of plants in the* Simaroubaceae* family is a group of alkaloids known as quassinoids that gives out its distinct bitter taste.* Azadirachta* sp. (Neem) on the other hand is intensively employed as a folklore remedy for a wide spectrum of diseases in India including in the treatment of cancer [7]. This plant also possesses insecticidal [8] and antibacterial activities [9].* Typhonium* sp. is a herbal plant that is locally known as* keladi tikus* or rodent tuber by the English. The tuber of this plant is used in combating a range of cancerous cell activities [10].

The aim of this study was to investigate the effect of* Brucea* sp.,* Azadirachta* sp., and* Typhonium* sp. extracts on the proliferation of cancerous oral cell lines KB and ORL-48. Responses of the cancer cells to the extracts were monitored and periodically captured for examination under an inverted microscope. Immunohistochemistry (IHC) fluorescent staining of extract-treated cells was performed as an indication of the mode of cell death, and the pattern of their fragmented DNA was produced for verification of the activity.

## 2. Methods

### 2.1. Preparation of Plant Materials

Fresh seeds of* Brucea javanica*, tubers of* Typhonium flagelliforme*, and leaves of* Azadirachta indica* were scientifically identified by a botanist and the voucher specimens were deposited at the Herbarium of Rimba Ilmu Botanical Garden, University of Malaya. The voucher specimen number of* Azadirachta indica* is KLU 47778. The references for the other two plants are however still under preparation.

100 g of each specimen was cleaned in running water and oven-dried at 37°C for 24 hours. The dried specimens were homogenized in distilled water at a ratio of sample to water of 1 : 10. The homogenate was boiled to one-third of the original volume and filtered through a filter paper (Whatman no. 1) to remove debris before it was further boiled to a final volume of 100 mL. The decoction was freeze-dried (EYELA FDU-1200, Tokyo) overnight and the powder obtained was stored in a dry cabinet for use in the study. Stock solution of the extract at the concentration of 100 mg/mL was prepared and diluted to the concentrations required for the respective experiments. The extract was sterilised by filtration using 0.2 *μ*m nylon syringe filter (Milipore, USA).

### 2.2. Preparation of Cell Lines

Two cancerous oral mucosal cell lines were used in the cytotoxic assay. KB cells were purchased from the American Type Cell Culture (ATCC, USA) while ORL-48 cells were obtained from the Cancer Research Initiatives Foundation (CARIF, Malaysia). ORL-48 cell line was developed from a female patient with gum tumour [11]. Fibroblast cell line was developed from an explant of gingival tissue scraped from an extracted tooth at the faculty's polyclinic. The cell line was used as a control to represent normal oral mucosa cells for comparative purposes.

### 2.3. Assessment of Antiproliferative Activity

The antiproliferative activity of the respective plants extracts was assessed based on a colorimetric assay using neutral red dye [12]. KB and ORL-48 cells at the concentration of 3 × 10^4^ cells/mL were dispensed into a 96-well culture plate (Nunc, Germany). A 20 mg/mL stock extract of each plant was diluted to varying concentrations of 0.1, 1, 10, 50, and 100 *μ*g/mL in separate wells using DMEM medium containing 10% fetal bovine serum, 1% penicillin-streptomycin, and amphotericin B. The cultures were incubated in a humidified incubator over a period of 72 hr at 37°C and 5% CO_2_ (Thermo Forma, USA). Wells containing cells in the absence of the extracts represented the negative control for the test. Following incubation, the culture medium was discarded and replaced with 100 *μ*L neutral red (1% v/v). The culture plates were further incubated for 2 hours after which the cells were washed with 1 mL of solution containing 1% sodium dodecyl sulfate. The culture plates were placed on a rocker (Nunc, Germany) for 30 min and the density of the detached viable cells that absorbed the red dye was assessed based on the optical absorbance read using an ELISA microplate reader (Bio Tek, USA) at a wavelength of 540 nm. The concentration of extract causing 50% of cell death known as the inhibition concentration (IC_50_) was determined by a graph of percentage of cell death versus concentrations of the plant extracts. The experiment was repeated three times in triplicate (*n* = 9) [13]. Similar procedure was carried out on normal fibroblast cells for comparative purposes.

### 2.4. Assessment of Cell Morphology in Response to the Extracts

Changes to the morphology of the cells in response to treatment by the respective extracts were monitored and periodically captured and analysed. A workstation for the analysis was set up encompassing a direct heat CO_2_ incubator (Thermo Forma), an inverted microscope (Olympus CK40), and a live resolution digital microscope (Moticam 2300). A concentration of 3 × 10^5^ cells was dispensed into 6-well titre plates, and 200 *μ*L of the respective plant extracts at concentrations within the range of 0.1, 1, 10, 50, and 100 *μ*g/mL was added. The proliferation of cells under the influence of the respective extracts was closely monitored at specific incubation period of 0, 3, 6, 9, 12, 24, 48, and 72 hr following the addition of the extract. Responses of the proliferating cells upon exposure to the extracts were viewed and captured under an inverted microscope. Images of cells captured at the 0 hr represented the control for the experiment.

### 2.5. Immunohistochemical (IHC) Staining: TUNEL Assay

The terminal deoxynucleotidyltransferase-mediated dUTP nick end labelling (TUNEL) technique was used to detect the presence of apoptotic cells. KB and ORL-48 cells (1.0 × 10^5^ cells) were seeded into 60 mm culture dishes with cover slips.* Brucea* sp.,* Typhonium* sp., and* Azadirachta* sp. at a concentration of 100 *μ*g/mL were added and the cells were incubated in a CO_2_ incubator for 72 hr. Following incubation, the cells were centrifuged at 4°C and the media removed. The cells were resuspended in 4% formaldehyde/PBS to a density of 1 × 10^6^ cell/mL, left to stand for 10 min at room temperature, pelleted down by centrifugation, and then resuspended and fixed in 80% ethanol. 1 mL of the suspension was transferred into a fresh microfuge tube and centrifuged. Following the removal of ethanol, 200 *μ*L of TBS (Tris buffered saline) was added and left to stand for 10 min before they were pelleted down and the TBS removed. The cells were resuspended in 100 *μ*L of 20 *μ*g/mL proteinase K and left to stand at room temperature for 5 min before they were pelleted down by centrifugation.

The cells were then resuspended in 100 *μ*L of TdT equilibration buffer, incubated at room temperature for 10–30 min, centrifuged, and resuspended in 60 *μ*L of TdT labelling reaction mixture. Following gentle mixing, the mixture was incubated in the dark at 37°C for 1–1.5 hr. Following centrifugation, the reaction mixture was discarded and the cells were resuspended in 200 *μ*L of TBS. The cells were washed, centrifuged, and examined under a fluorescence microscope.

### 2.6. DNA Fragmentation Analysis

Gel electrophoresis was carried out to determine the band pattern of DNA fragments from KB and ORL-48 extract-treated cells. The Suicide-Track DNA Ladder Isolation kit that uses a nonisotopic method for the detection of DNA laddering in monolayered cells was used.

#### 2.6.1. Collection of DNA

Cells cultured in 6-well titre plates for 48 hours were detached by the addition of accutase (PAA, Austria). Following centrifugation at 9,100 ×g, the cell pellet was resuspended to a concentration of 5 × 10^5^ to 1 × 10^6^ cells/mL using a haemocytometer. Prior to electrophoresis, the cells were centrifuged and resuspended in 55 *μ*L of solution #1 (Kit component no. JA1825-1.38ML) followed by the addition of 20 *μ*L of solution #2 (Kit component no. JA1826-.5ML). Following an hour incubation at 37°C, 25 *μ*L of solution #3 (Kit component no. JA1827-.625ML) was added, gently mixed, and reincubated overnight at 50°C. 2 *μ*L of Pellet Paint Co-Precipitant (Kit component no. JA1836-0.05ML), 60 *μ*L of 3 M sodium acetate, and 662 *μ*L of 2-propanol were then added. Following gentle mixing, the microvial was left to stand at room temperature for 2 min or until a pink pellet was clearly visible at its bottom. The microvial was centrifuged at 15,000–16,000 ×g for 5 min, and the cell pellet was rinsed twice with 500 *μ*L of 70% ethanol followed by 500 *μ*L of 100% ethanol. Following centrifugation, the final cell pellet was collected, air dried to remove excess ethanol, and resuspended in 50 *μ*L of resuspension buffer.

#### 2.6.2. Gel Preparation

Agarose gel (1.5%) of 0.75 cm thick was prepared in TBE (Tris/borate/EDTA) with the addition of 0.5 mg/mL of ethidium bromide. The agarose mixture was poured into an electrophoresis chamber, and a gel comb was inserted to create wells for samples. Once solidified, the gel was transferred into a gel buffer tank. 5 *μ*L of DNA ladder samples in gel loading buffer was carefully loaded into the wells, and 5 *μ*L of 100 bp lab DNA ladder was used as a marker. The electrophoresis was run at a constant ~50 volts until the dye front has reached 1-2 cm from the bottom of the gel. The gel was then examined through UV illumination for the detection of DNA products of the cancer cells.

### 2.7. Statistical Analysis

Statistical analyses were performed using the SPSS version 11.5 software. The distributions of data were evaluated using nonparametric tests, and the results were considered statistically significant if the *P* value < 0.05 from two-sided tests.

## 3. Results

### 3.1. Antiproliferative Activity

Based on the IC_50_ value, the extract of* Brucea* sp. exhibited antiproliferative activity on KB cells at 24.37 ± 1.75 *μ*g/mL while some inhibition by* Typhonium* sp. and* Azadirachta* sp. was observed at 95.67 ± 1.15 *μ*g/mL and 80.75 ± 6.01 *μ*g/mL, respectively (Table 1).* Brucea* sp. also exhibited strong potency against ORL-48 cells at 6.67 ± 1.15 *μ*g/mL. The other two extracts showed no effect on the proliferation of ORL-48 cells.

### 3.2. Morphological Changes of Cancer Cells in Response to Extracts

**Figure 1:**
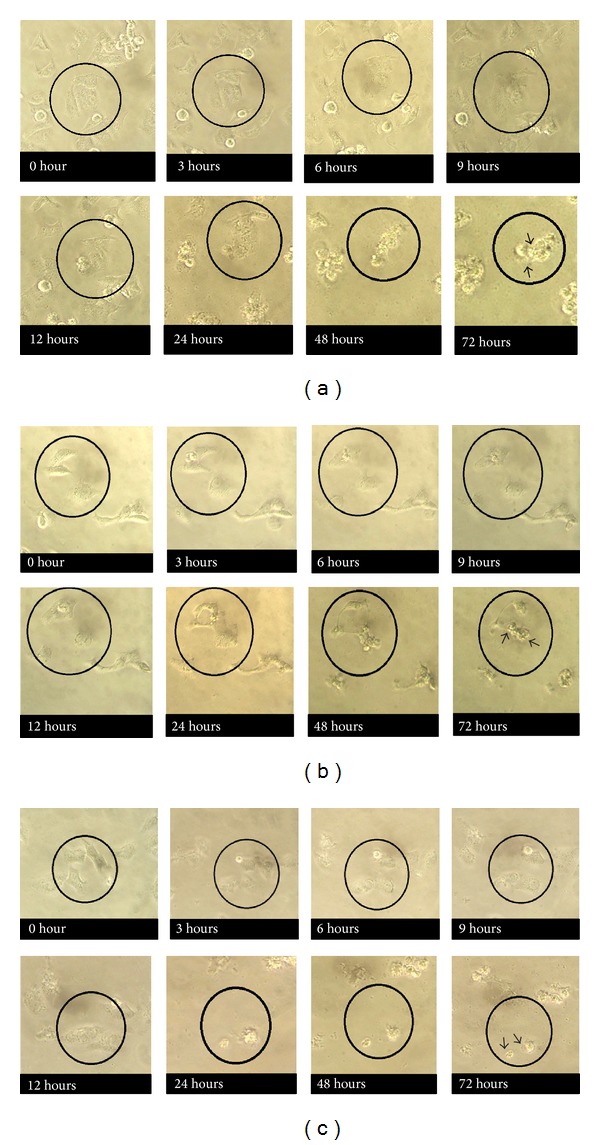
Responses of KB cells to 100 *μ*g/mL extract of (a)* Brucea *sp., (b)* Azadirachta* sp., and (c)* Typhonium *sp. monitored from 0 hr to 72 hr following treatment. Sign of cell attrition was focused on cells within the black circles. The arrow heads pointed at the apoptotic cells. Images were obtained using an inverted microscope. (40x).

**Figure 2:**
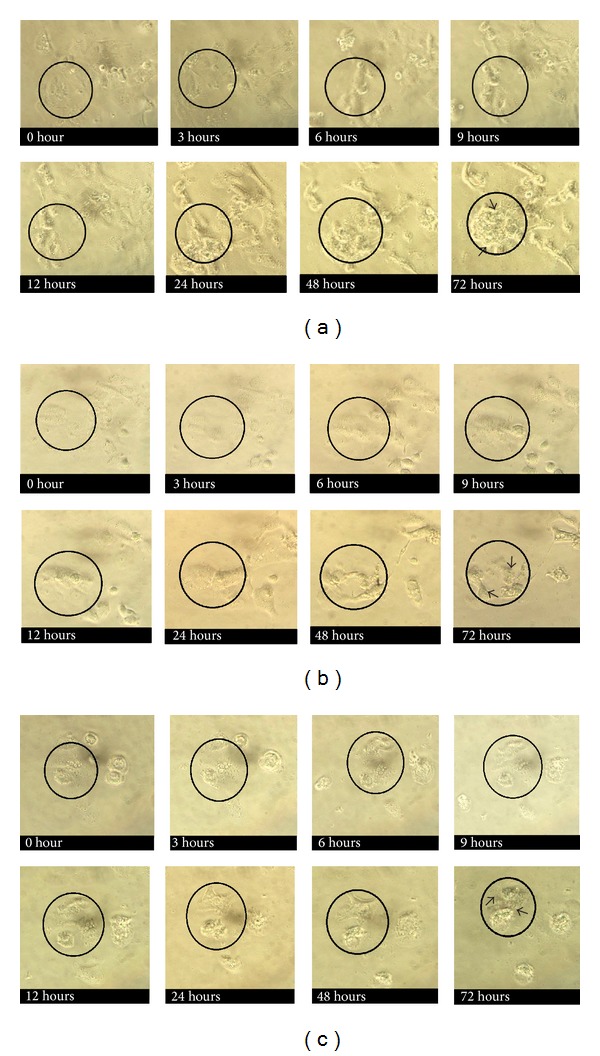
Responses of ORL-48 cells to 100 *μ*g/mL extract of (a)* Brucea* sp., (b)* Azadirachta* sp., and (c)* Typhonium* sp. monitored from 0 hr to 72 hr following treatment. Sign of cell attrition was focused on cells within the black circles. The arrow heads pointed the apoptotic cells. Images were obtained using an inverted microscope. (40x).

Obvious changes to KB and ORL-48 cells were observed at 24 hr of incubation following treatment with 100 *μ*g/mL of* Brucea* sp. extracts. The black circles marked the area in which the cells were observed throughout a 72 hr incubation period. Morphological changes include reduction in the size of the cells. The cells gradually become flat and shrunken with the appearance of small vesicle bodies (apoptotic bodies). Similar observations were also made when the cells were treated with 100 *μ*g/mL of* Azadirachta* sp. and* Typhonium* sp. However, the first sign of apoptotic activity was observed much later following treatment at 72 hr of incubation (Figures 1 and 2).

### 3.3. TUNEL Assay

**Figure 3:**
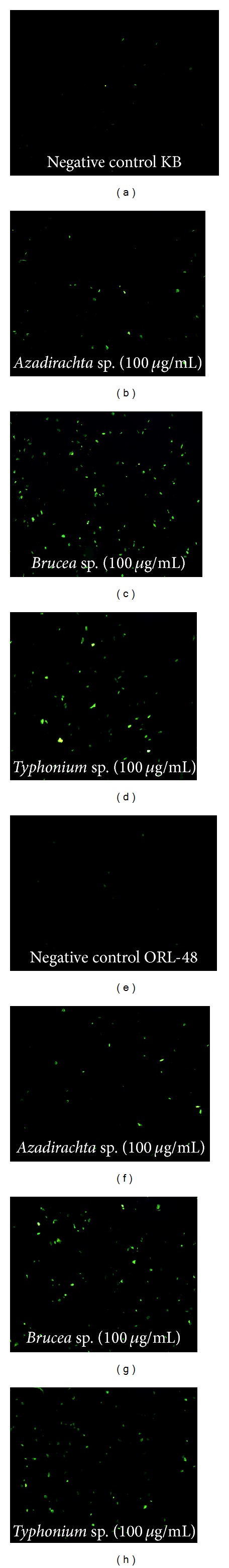
Images of fluorescent-stained KB and ORL-48 cells following treatments with 100 *μ*g/mL of* Azadirachta* sp. ((b), (f)),* Brucea *sp. ((c), (g)), and* Typhonium *sp. ((d), (h)) at 10x magnification. (a) and (b) were the negative controls for KB and ORL-48 cells, respectively.

Green fluorescent spots were present in all the images of KB and ORL-48 extract-treated cells (Figure 3). These observations were indicative of the apoptotic activity of* Brucea* sp.,* Azadirachta* sp., and* Typhonium* sp. extracts on both cancer cells. This IHC assay was employed to confirm the apoptotic activity induced by the respective extracts on KB and ORL-48 cells. Apoptotic KB or ORL-48 cells appeared as fluorescence bright green spots in the images, while nonapoptotic cells were round and appeared as faint dull green or red spots that were rather difficult to visualize (Figure 3). Antimomycin D was used as a positive control in the experiment (see Supporting Figure  1 in Supplementary Material available online at http://dx.doi.org/10.1155/2014/125353).

### 3.4. DNA Fragmentation Analysis

**Figure 4:**
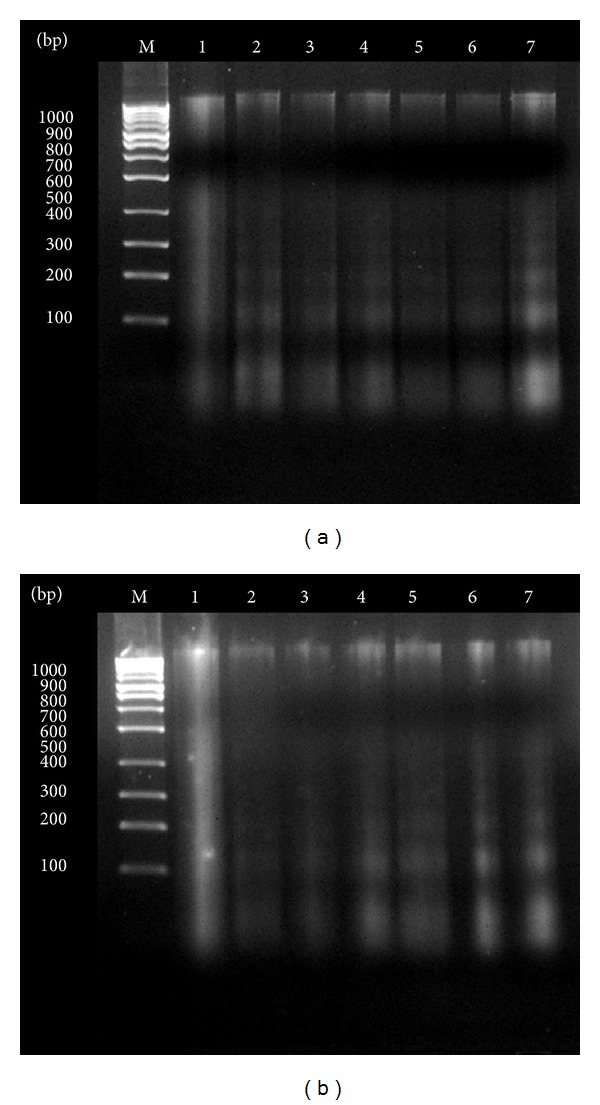
DNA band patterns of (a) KB and (b) ORL-48 cells treated with various concentrations of* Brucea* sp. Lane 1: negative control; Lanes 2 to 7 were bands of cancer cells treated with 1.0, 10.0, 25.0, 50.0, 75.0, and 100.0 *μ*g/mL extract of* Brucea* sp.

Induction of apoptosis on KB and ORL-48 cells by* Brucea* sp.,* Azadirachta* sp., and* Typhonium* sp. extracts was validated by DNA fragmentation analysis using gel electrophoresis technique. The DNA bands obtained from both* Brucea* sp. extract-treated KB and ORL-48 produced ladder pattern as observed from Lane 2 to 7 (Figure 4). A ladder formation was used to indicate that the DNA has undergone fragmentation, and each fragment corresponded to a band in the ladder. A similar DNA banding pattern but of lower intensity was also observed on KB and ORL-48 cells following treatment with the extracts of* Typhonium* sp. and* Azadirachta* sp. (Supporting Figures 2 and 3).

## 4. Discussion

The ability of cancerous cell populations to expand in number is determined by their ability to proliferate and, in many cases, to form tumour. Three plants species with reputed anticancer potentials among the local practitioners were selected for this research. Other than topical application of paste prepared from these plants to the tumour area, consumption of decoctions prepared from the plants is also being practiced. Despite their popular usage as local medicines, information on the exact formulation and amount of plant specimen used in the treatments is not standardised. Results obtained from this study revealed information with regards to the ability of aqueous extracts of* Brucea* sp.,* Typhonium* sp., and* Azadirachta* sp. to inhibit the proliferation of cancer cell lines originating from the oral mucosa.

Antiproliferative assessment showed that the proliferation of both KB and ORL-48 cells was effectively inhibited by the extract of* Brucea* sp. with activity on the latter cells being more prominent (Table 1).* Brucea* sp. induced morphological effects on both KB and ORL-48 at 24 hr of treatment compared to at 72 hr for* Azadirachta* sp. and* Typhonium* sp. Among the signs of cells going through attrition process is the appearance of smaller, flattened, and shrunken cells (Figures 1 and 2). These are among the characteristics of cells undergoing apoptosis. Cell death can be achieved via apoptosis as well as necrosis. In the search for active compounds with anticancer activity, an agent that induces cell death via apoptosis is preferred [2].

Apoptosis is a programmed cell death that removes or eliminates targeted unwanted or dead cells. Other than shrunken cells, characteristics of apoptotic cells include condensation of the cytoplasm and nucleus, aggregation of chromatin, and formation of membrane-bound vesicles known as apoptotic bodies [14]. Necrosis on the other hand refers to a pathological activity. Necrosis is known to be proinflammatory and is marked by swelling of the cell that is often accompanied by chromatin condensation. Necrotic cells eventually experienced cellular and nuclear lysis along with subsequent inflammation [15], which would be unfavorable for an anticancer agent. In this study, results obtained from the IHC staining procedure suggested the apoptotic activity of* Brucea* sp.,* Typhonium* sp., and* Azadirachta* sp. (Figure 3). Green fluorescent spots indicating the presence of apoptotic cells were observed when the extract-treated cells were examined under a fluorescent microscope (Figure 3). As anticipated with the strong antiproliferative activity of* Brucea* sp., the population of green spots in images of* Brucea*-treated cells was higher compared to cells treated with the other two extracts.

Verification of the apoptotic activity of* Brucea* sp. was carried out based on the pattern of DNA bands produced from a gel electrophoresis. In apoptosis, cells are lysed gradually and systematically to produce membrane-bound apoptotic bodies, which was suggested to play a major role in suppressing inflammatory responses to other neighbouring cells. Apoptotic bodies or cells which underwent apoptosis produce a specific pattern of DNA fragments with the multiples of 200 bp due to specific action of activated nucleases [16]. These isolated fragments produced bands in a ladder pattern, in contrast with the smeared pattern produced from necrosis activity (Figure 4). Results obtained in this study in a way supported the various claims made by researches on the anticancer properties of this plant [17–19]. Further studies are being carried out to identify the active principle of* Brucea* sp. extract. Thus, it can be concluded that the strong antiproliferative activity of* Brucea* sp. extract on oral cancer cells suggests its possible development as an anticancer agent. The mode of action of* Brucea* sp. was by the induction of apoptotic activity on cancer cells.

## Supplementary Material

Description for Supplementary 1: Cells undergoing apoptotic activity eventually produces fragments of dense granular particles due to the activities of endogenous nucleases. These apoptotic bodies are easily stained green fluorescent using the IHC technique. Actinomycin D is an alkylating agent that induces apoptosis of cancer cells and was thus used as a positive control in the study.
Description for Supplementary 2 & 3: Apoptosis of cancer cells resulted from DNA fragmentation of the chromatin into nucleosomal units. When run on agarose gel electrophoresis these units appear as DNA ladder. Thus, determining whether a cell exhibits DNA fragmentation can provide information about the mode of cell death induced by the respective extracts.

## Figures and Tables

**Table 1:** The IC_50_ of *Brucea *sp., *Azadirachta *sp., and *Typhonium *sp. extracts on KB and ORL-48 cancer cells. Fibroblasts represented as normal cells in the study.

Extracts	Antiproliferative activity, IC_50_ (*μ*g/mL)		
	Fibroblasts	KB cells	ORL-48 cells
*Brucea *sp.	ND	24.37 ± 1.75	6.67 ± 1.15
*Azadirachta *sp.	ND	95.67 ± 1.15	>100
*Typhonium *sp.	ND	80.75 ± 6.01	>100

ND: not determinable.
