# Supplementary material for: Apoptosis-Inducing Effect of Three Medicinal Plants on Oral Cancer Cells KB and ORL-48
Source: ScientificWorldJournal. 2014 Jul 24;2014:125353. doi: 10.1155/2014/125353 (PMC4134791; doi:10.1155/2014/125353)
Supplement: Supplementary file 1 — Description for Supplementary 1: Cells undergoing apoptotic activity eventually produces fragments of dense granular particles due to the activities of endogenous nucleases. These apoptotic bodies are easily stained green fluorescent using the IHC technique. Actinomycin D is an alkylating agent that induces apoptosis of cancer cells and was thus used as a positive control in the study. Description for Supplementary 2 & 3: Apoptosis of cancer cells resulted from DNA fragmentation of the chromatin into nucleosomal units. When run on agarose gel electrophoresis these units appear as DNA ladder. Thus, determining whether a cell exhibits DNA fragmentation can provide information about the mode of cell death induced by the respective extracts. [file 125353.f1.pdf]

(A)

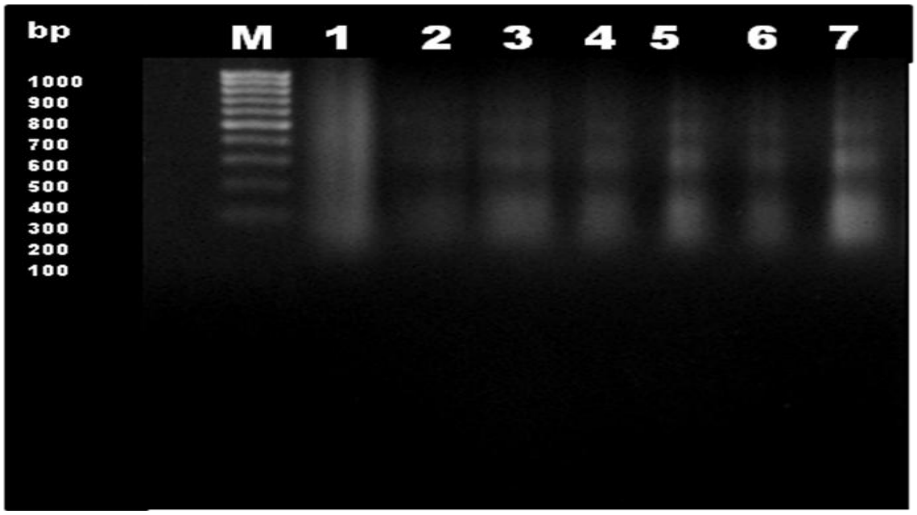

(B)

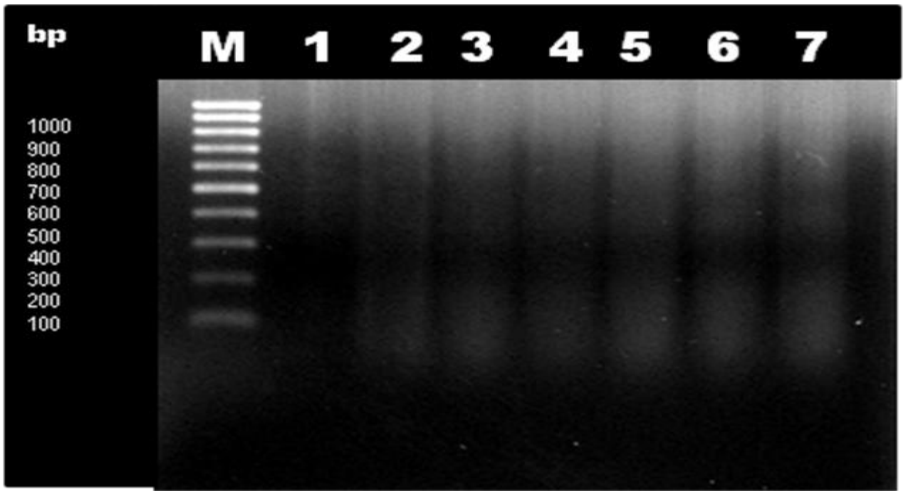

Supporting 1: Expression of DNA fragments from KB (A) and ORL-48 (B) cells after treatment with various concentrations of *A. indica* extract. Lane 1, negative control; Lane 2-7, cancer cells treated with 1.0, 10.0, 25.0, 50.0, 75.0 and 100.0 µg/mL of *A. indica* extract, respectively.

(A)

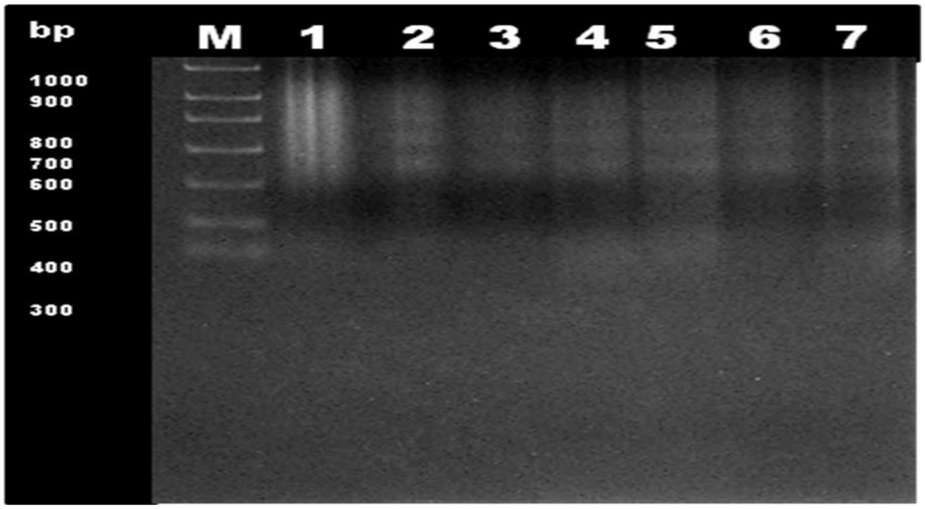

(B)

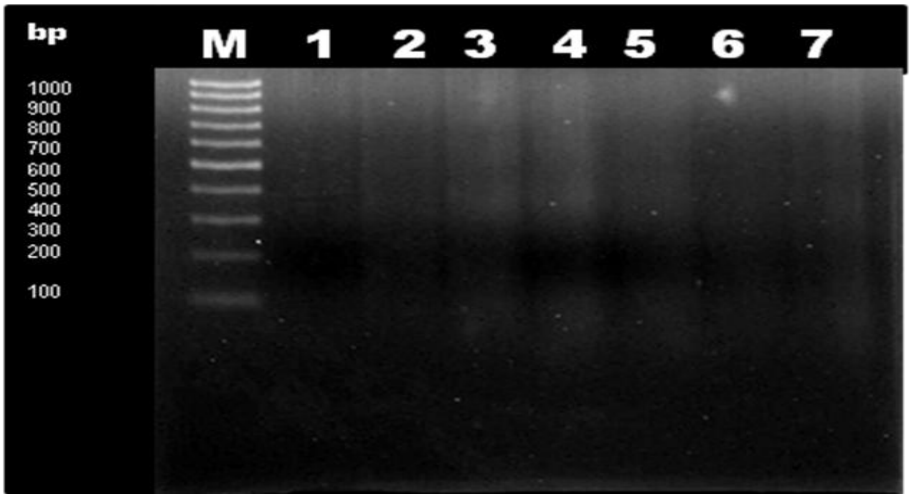

Supporting 2: Expression of DNA fragments from KB (A) and ORL-48 (B) cells after treatment with various concentrations of *T. flagelliforme* extract. Lane 1, negative control; Lane 2-7, cancer cells treated with 1.0, 10.0, 25.0, 50.0, 75.0 and 100.0 µg/mL of *T. flagelliforme* extract, respectively.

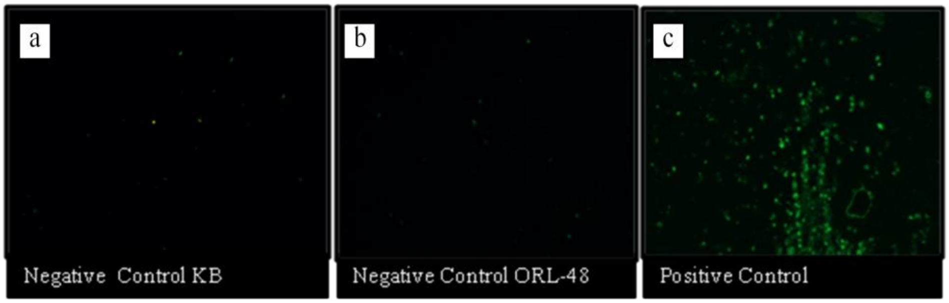

Supporting 3: Images of untreated KB (a) and ORL-48 (b) cells to represent as negative control and (c) actinomycin D-treated cells to represent as positive control. Observed the presence of green fluorescent spots showing the presence of apoptotic cells following treatment with Actinomycin D. (10x magnification).
